# Development of directed global inhibition, competitive inhibition and behavioural inhibition during the transition between infancy and toddlerhood

**DOI:** 10.1111/desc.13193

**Published:** 2021-12-07

**Authors:** Alexandra Hendry, Isobel Greenhalgh, Rhiannon Bailey, Abigail Fiske, Henrik Dvergsdal, Karla Holmboe

**Affiliations:** ^1^ Department of Experimental Psychology University of Oxford Oxford UK; ^2^ Department of Psychology University of Cambridge Cambridge UK; ^3^ School of Psychology University of Surrey Guildford UK; ^4^ Division of Entrepreneurship and Innovation Nord University Business School Bodø Norway; ^5^ School of Psychological Science University of Bristol Bristol UK

**Keywords:** behavioral inhibition, executive function, infant, inhibitory control, self‐regulation, toddler

## Abstract

Inhibitory control (IC) is a core executive function integral to self‐regulation and cognitive control, yet is itself multi‐componential. Directed global inhibition entails stopping an action on demand. Competitive inhibition is engaged when an alternative response must also be produced. Related, but not an executive function, is temperamentally‐driven wariness of novelty, known as behavioural inhibition. Understanding early development of these components has been hampered by a shortage of suitable measures. We combine established and novel measures to capture directed global inhibition (Toy Prohibition, Touchscreen Prohibition), competitive inhibition (A‐not‐B, Early Childhood Inhibitory Touchscreen Task; ECITT) and behavioural inhibition (Touchscreen Approach) in 113 10‐ and 16‐month‐olds (73 seen longitudinally). ECITT performance shows good 1‐week test‐retest reliability at 10‐months (*r *= 0.30–0.60) but little stability to 16‐months. Directed global inhibition performance shows developmental progression but little stability of individual differences from 10 to 16 months. Performance on measures targeting similar IC components shows greater coherence at 16‐months (*r *= 0.23–0.59) compared with 10‐months (*r *= 0.09–0.35). Probing of ECITT condition effects indicates toddlers are more able, compared with infants, to override immediate prepotencies; indicative of increasingly flexible control over behaviour. However, exerting IC over cumulative prepotencies appears just as challenging for toddlers as infants. Exploratory analyses show little evidence for cross‐sectional or longitudinal associations between behavioural, directed global and competitive inhibition. In combination, these findings indicate that IC is not yet a stable, unidimensional construct during the transition between infancy and toddlerhood, and highlight the need for careful selection of multiple measures for those interested in capturing early variation in IC.

## 1 INTRODUCTION

Inhibitory control (IC) entails stopping or redirecting a thought or action, often in favour of an alternative response. IC thus enables us to resist temptation and withhold actions that are risky or no longer appropriate to a situation. Difficulties with IC are common amongst children born pre‐term (van Houdt et al., 2019) and have been associated with poor academic outcomes (Allan et al., 2014), ADHD (Pauli‐Pott & Becker, 2011), and externalising problems more generally (Schoemaker et al., 2013). Understanding more about the development of IC in infancy may lead to advances in early intervention to support children at risk for later difficulties.

### 1.1 Facets of inhibitory control: Directed global inhibition and competitive inhibition

Although in common parlance, and in many cognitive studies, IC is often treated as a homogenous construct, it may be more useful to consider the distinct types of inhibitory effects that are elicited by different contexts or task demands. For example, the requirement to stop an action on demand, such as in response to an external prohibition (“Don't touch!”) can be considered to exert different demands than the requirement to execute an alternative response to a withheld action (e.g., pulling rather than pushing the one door in your office building which opens in that direction). Munakata et al. (2011) have suggested that these two effects—labelled, respectively, directed global inhibition, and competitive inhibition—are associated with different neural pathways. Accounting for this distinction may be crucial for understanding the mechanisms of IC, how it develops, and under what circumstances IC may be impaired or improved.

Conceptualising directed global inhibition and competitive inhibition as distinct processes does not preclude the possibility of interaction between the two, as we discuss in more detail below.

### 1.2 Directed global inhibition and competitive inhibition amongst toddlers and pre‐schoolers

The majority of research into the development of IC focuses on children aged 2 years and older (Petersen et al., 2016). In studies with toddlers and pre‐schoolers, directed global inhibition is generally measured with prohibition or delay tasks, whereby the participant is instructed not to touch a desirable object, or to wait until a particular signal is given. Competitive inhibition tasks tend to involve either inhibiting a reaching response to a well‐primed (i.e., prepotent) location in favour of an alternative location (Holmboe et al., 2021) or reversing a previously‐established stimulus‐response mapping (i.e., saying “day” when shown a picture of a moon, and “night” when shown a picture of a sun) (Gerstadt et al., 1994). Such tasks are sometimes called complex response inhibition or conflict inhibition tasks (Garon et al., 2008; Nigg, 2000; Petersen et al., 2016).

Studies have demonstrated dramatic improvements in IC across the toddler and pre‐school years (Garon et al., 2008; Petersen et al., 2016), but also stability in individual differences in IC; particularly for tasks involving competitive inhibition (Carlson et al., 2004; Holmboe et al., 2021; Hughes & Ensor, 2007; Kloo & Sodian, 2017; Willoughby et al., 2012). Longitudinal studies indicate that coherence of performance across different types of IC increases between ages 2 and 3 years, as evidenced by age‐related increases in inter‐correlations between measures (Carlson et al., 2004; Kochanska et al., 2000). However, some of this change may be attributed to non‐inhibitory task demands (such as language comprehension or motor coordination) influencing performance to a lesser extent for older children. One limitation of current research is that pre‐school competitive inhibition tasks often involve working memory demands in addition to inhibitory demands (Garon et al., 2008; Petersen et al., 2016), making it difficult to interpret whether low coherence between directed global inhibition and competitive inhibition tasks is due to a genuine differentiation of inhibitory processes, or to differences in working memory demands between tasks. Further, a lack of tasks which can be used with infants as well as toddlers has to date limited our understanding of the early development of IC (Holmboe et al., 2021; Petersen et al., 2016).

**RESEARCH HIGHLIGHTS**

1. Demonstrates that directed global inhibition and competitive inhibition are dissociable aspects of inhibitory control, even in infancy.
2. Presents performance data on new touchscreen measures of directed global inhibition and competitive inhibition collected from 10‐ to 16‐month‐olds.
3. Considers the role of behavioural inhibition in influencing performance on inhibition tasks.
4. Provides insights into developmental progression, longitudinal stability and cross‐component associations involved in the early development of inhibitory control.

### 1.3 Directed global inhibition and competitive inhibition in infancy

Research into the development of IC in infancy is constrained by babies’ more‐limited response repertoires, and their inability to follow complex instructions. Nevertheless, there is some indication that infants are able to exercise both global inhibition and competitive inhibition within the first year of life (Diamond, 1985; Kochanska et al., 1998). Directed global inhibition in infancy can be measured with variants of the prohibition (“Don't touch”) paradigm, in which the caregiver or experimenter prohibits the infant from touching an attractive object and the latency to touch the object is recorded. Although toddlers improve dramatically in their ability to exercise restraint on this type of task during the second and third years of life (Friedman et al., 2011), performance at 12 months shows some stability to ages 18 and 24 months (Frick et al., 2019).

A limitation of research into directed global inhibition in infancy is that commonly only a single‐trial prohibition task is used, which increases vulnerability to measurement noise. Repeating the task using the same or similar target object may lead to learning effects, or to confusion or distress for the infant. However, by changing the surface characteristics of the target sufficiently—e.g., by switching from a physical object to another enticing stimulus, such as an animation on a touchscreen device—it may be possible to elicit directed global inhibition twice rather than once. Individual differences in performance on a touchscreen prohibition task would be expected to show moderate‐to‐high associations with individual differences in performance on an object prohibition task (allowing for the measurement noise inherent in both tasks): but to our knowledge this is yet to be empirically tested.

Competitive inhibition is most commonly measured in infancy with the A‐not‐B task. In this task the researcher places a desirable object in one of two identical covered wells (“A”) then allows the infant to retrieve (often after a delay, which may be lengthened to increase task demands) the object from that location. This action is repeated in order to build up the infant's prepotent reaching response to A. The object is then hidden in the alternate location (“B”) such that a new response (reach to “B”) must be initiated, and the competing prepotent response inhibited. At 9 months, variation in infants’ performance on the canonical version of the A‐not‐B task (in which a “B” trial follows two successful “A” trials) may be attributable to individual differences in IC, attentional focus and working memory (Bell & Adams, 1999; Diamond, 1985; Holmboe et al., 2018), as well as to non‐inhibitory processes such as motor control (Clearfield et al., 2006). Although developmental transitions in motor control may mask long‐term stability in A‐not‐B performance across infancy, secondary analysis of published data (Cuevas & Bell, 2010) reveals small‐to‐moderate correlations on a month‐by‐month basis on reaching and looking versions of the A‐not‐B task between the ages of 6 and 10 months (Martha Ann Bell, personal correspondence).

To date, there has been limited research into associations between individual differences in A‐not‐B performance and other measures of IC in infancy. Devine et al. (2019) found no significant association between directed global inhibition and performance on a variant of the A‐not‐B task amongst 14‐month‐olds—however, in this variant, trials were completed in a fixed order regardless of whether a prepotent response was established, potentially limiting the competitive inhibition demands of the task. Miller and Marcovitch (2015) found no significant association between compliance during an extended prohibition task and A‐not‐B performance amongst 14‐ and 18‐month‐olds—but used a multi‐location version of the task which may have been more sensitive to variation in working memory than to IC. Using a canonical version of the A‐not‐B task, Holmboe et al. (2008, 2018) did find significant associations between A‐not‐B performance and performance on a saccadic inhibition task at 9 months, but as no measure of directed global inhibition was included in those studies, it remains to be tested whether performance on directed global inhibition and competitive inhibition tasks show coherence during infancy and early toddlerhood.

Recently, a new measure of competitive inhibition has been developed; the Early Childhood Inhibitory Touchscreen Task (ECITT) (Holmboe et al., 2021). Like the A‐not‐B task, the ECITT elicits competitive inhibition by first establishing a prepotent reaching response to one location (a target to one side of a touchscreen), and then requiring that the prepotent response be inhibited in favour of a reach to the contra‐lateral location. A key difference from the A‐not‐B task is that the target location is visually‐cued (by a smiley‐face icon) throughout the test part of each trial: therefore, working memory demands are minimal. In contrast, even in the “immediate” retrieval version of the A‐not‐B task, the participant must hold in mind the target's location in the interim period between the reward being hidden and them being allowed to reach for it.

The ECITT has a further advantage over the A‐not‐B task in that, being administered via touchscreen, it is particularly easy to standardise administration (i.e., duration and order of rewards and number of trials). As only a touchscreen is needed for administration of the ECITT, there are also fewer props involved in comparison to the A‐not‐B task, which means less likelihood of the infant becoming distracted. Further, piloting has demonstrated that infants find the task engaging, and the short trial durations (as low as 4 s, depending on infants’ response speeds) mean that a relatively high number of trials can be administered before the infant becomes fatigued, which in turn allows for greater sensitivity to individual differences. Finally, because the researcher simply holds the touchscreen and is not actively involved in the test or reward parts of the trials, the ECITT has low social demands in comparison to the A‐not‐B task—where the researcher administers the hiding events and also tells the participant whether they were correct or not. This reduces the potential for pragmatic misinterpretation (Topál et al., 2008).

The ECITT was designed specifically with a large age‐range in mind and although performance on the ECITT improves significantly between 18 and 30 months of age, younger toddlers do not show floor effects on the task (Holmboe et al., 2021). Amongst adults and children aged 6 and over, performance on the advanced version of the ECITT correlates with performance on another well‐established IC measure (the Stop‐signal task). It is yet to be demonstrated whether the ECITT elicits competitive inhibition in infants under 18 months, or whether individual differences in ECITT performance in infancy show coherence with other measures of IC.

### 1.4 Development of directed global inhibition and competitive inhibition: Competing models

As yet, the developmental pathways involved in directed global inhibition and competitive inhibition are unclear, and there are a number of competing, possible models. Influential work by Garon et al. suggests that executive function development proceeds in a hierarchical manner, with simple skills preceding, and supporting, the development of complex skills. Intuitively, in line with this model, we might expect that directed global inhibition skills precede competitive inhibition skills, and that infants with high directed global inhibition performance scores would tend to achieve higher competitive inhibition performance scores at later time‐points. Directed global inhibition—competitive inhibition associations could also apply concurrently: For example, older children and adults may use directed global inhibition skills to deliberately slow their responding in order to dissipate prepotencies in competitive inhibition tasks (Simpson & Carroll, 2019). Amongst infants, slowing is unlikely to be a deliberate strategy, but may nevertheless be a mechanism by which an infant who is more‐able to control their impulses reduces competitive inhibition demands and thereby performs more‐accurately on such tasks. In this case, we might expect that infants with higher directed global inhibition skills also achieve higher scores on competitive inhibition tasks at the same time‐point.

Another possibility is that competitive inhibition and directed global inhibition skills follow distinct developmental pathways, achieving some level of integration only later on in childhood. Under this model, we might expect associations between measures of competitive inhibition and directed global inhibition skills to be low.

### 1.5 Behavioural inhibition as an additional influence on inhibitory control performance

As well as directed global and competitive inhibition, behavioural inhibition may also contribute to performance on IC tasks. Behavioural inhibition entails a reluctance to approach, or withdrawing from, an unfamiliar or daunting situation. Whereas IC allows us to exercise top‐down voluntarily control over our behaviour, behavioural inhibition is more reactive and involuntary (Derryberry & Rothbart, 1997). Behavioural inhibition can be considered a temperament trait, which shows some stability over time and context, as well as continuity during infancy and early childhood (Pérez‐Edgar & Guyer, 2014).

One way in which behavioural inhibition may boost performance on IC tasks is through dissipating prepotencies by introducing a delay. As previously noted, performance‐boosting delays during competitive inhibition tasks can be a top‐down strategy drawing on directed global inhibition, but they may also be somewhat inadvertent, resulting from a non‐impulsive, inhibited approach style which introduces a delay across all responses. Amongst 3‐ to 5‐year‐olds, behavioural inhibition is positively associated with performance on IC tasks (Eisenberg et al., 2013; Thorell et al., 2004). In terms of the *developmental* mechanisms linking behavioural inhibition and IC, Aksan and Kochanska (2004) present evidence to support the argument that the non‐impulsive (slower) approach style characteristic of inhibited children not only has immediate benefits on performance on directed global inhibition tasks at age 2–3 years, but may also facilitate long‐term development of competitive inhibition; however, these developmental associations have yet to be observed in infancy.

### 1.6 The current study

The current study aims to progress research into the development of IC in infancy in two inter‐connected ways. Firstly, we aim to broaden the repertoire of IC tasks that are available to infancy researchers. Specifically, our objective is to present a battery of short, engaging tasks that can be used to target directed global inhibition and competitive inhibition and (as a secondary objective) behavioural inhibition as potentially distinct constructs, and which are suitable for both infants and toddlers. This battery comprises both existing, commonly‐used tasks (the A‐not‐B task, and Toy Prohibition task) and newer tasks which have been developed to harness the possibility for rapid, scalable data collection afforded by developments in touchscreen technology (the ECITT, Touchscreen Prohibition, and Touchscreen Approach tasks). Our motivation is that increasing the number of age‐appropriate, theory‐driven measures of infant directed global inhibition and competitive inhibition will promote, in the long‐term, higher‐quality data collection and thereby improve the conceptual interpretations that can be made from such data. This leads to our second aim, which is to provide new insights into developmental progression, longitudinal stability and cross‐component associations involved in the early development of IC by presenting longitudinal data collected from 115 infants at ages 10 and 16 months using this battery of IC tasks. These insights are intended to be used as a first step to identifying which of the models of early IC development outlined above is most plausible, so that this may then be tested in larger‐scale follow‐up studies, using more advanced analytic approaches, such as Structural Equation Modelling.

#### 1.6.1 Research questions and associated hypotheses

Objective 1:

1. Is the Touchscreen Prohibition task suitable as a measure of directed global inhibition in infancy?
  - The Touchscreen Prohibition task elicits variation that can reasonably be attributed to directed global inhibition, and not behavioural inhibition, at 10 and 16 months; that is, the association between Touchscreen Prohibition performance and Touchscreen Approach performance is weak‐to‐null, whereas individual differences in performance on Touchscreen Prohibition and the Toy Prohibition task are positively correlated at 10 and 16 months.
2. Is the ECITT suitable as a measure of competitive inhibition in infancy?
  - The ECITT elicits condition effects that can reasonably be attributed to competitive inhibition, in that:
    ○ i) participants make significantly more errors in inhibitory trials than in prepotent trials at 10 months (pre‐registered) and 16 months.
    ○ ii) response times are significantly slower for inhibitory trials than prepotent trials at 16 months.

In addition, we will conduct exploratory tests to investigate whether condition effects are influenced by side biases.

b) Individual differences in ECITT performance at 10 months show test‐rest reliability (pre‐registered).
c) Individual differences in performance on the A‐not‐B and the ECITT are positively correlated at 10 months (pre‐registered) and 16 months (pre‐registered).

In addition, we will conduct exploratory tests to investigate whether condition effects are influenced by spill‐over effects from ECITT to A‐not‐B

Objective 2:

3. Do infants show developmental progression in terms of directed global inhibition and competitive inhibition between the ages of 10 and 16 months?
  - Latencies to touch on the Toy Prohibition and Touchscreen Prohibition tasks are longer at 16 months than at 10 months.
  - Performance scores on the ECITT task are higher at 16 months than at 10 months.
4. Do individual differences in performance on IC and behavioural inhibition measures show longitudinal stability between the ages of 10 and 16 months?
  - Latencies to touch on the Toy Prohibition and Touchscreen Prohibition tasks at 10 months are positively correlated with latencies to touch on the same task at 16 months.
  - Scores on the ECITT at 10 months are positively correlated with ECITT scores using the same variable at 16 months (pre‐registered).
  - A‐not‐B switching scores at 10 months are positively correlated with A‐not‐B switching scores at 16 months (pre‐registered).
  - Latency to touch on a behavioural inhibition task at 10 months is positively correlated with latency to touch on the same task at 16 months.
5. Is there evidence for associations between different components of IC, and between measures of IC and behavioural inhibition?
  - Latency to touch on a behavioural inhibition task is positively correlated with individual differences in performance on the ECITT (pre‐registered) and A‐not‐B tasks, at 10 and 16 months, cross‐sectionally.
  - Individual differences in performance on directed global inhibition tasks are positively correlated with individual differences in performance on competitive inhibition tasks at 10 and 16 months, cross‐sectionally (exploratory).
  - Individual differences in performance on directed global inhibition tasks at 10 months are positively correlated with individual differences in performance on competitive inhibition tasks at 16 months, and vice‐versa (exploratory).

Additionally, we also explored whether there were sex differences in performance on each of our measures in order to ascertain whether sex should be included as a covariate in any of the analyses.

Pre‐registered hypotheses are registered at https://aspredicted.org/wh44n.pdf and https://aspredicted.org/ef5sr.pdf.

## 2 METHOD

### 2.1 Participants

One hundred and fifteen infants were recruited between September 2017 and March 2019 via the University of Oxford BabyLab volunteer database and social media as part of a study investigating the development of executive functions; see Table 1 for sample demographics. Two participants were excluded from analysis due to oxygen deprivation at birth (*n *= 1) or being born before 37 weeks, with birth weight under 2.5 kg (*n *= 1). Of the remaining infants, 73 infants were seen at 10 and 16 months, 35 were seen at 10 months only, and five were seen at 16 months only. Of the 108 infants seen at 10 months (*M *= 10.18, *SD *= 0.38 months), 55 attended a second visit one week (*M *= 7.69, *SD *= 2.67 days) after their initial visit, where they completed the ECITT for a second time.

**TABLE 1:** Participant demographics

Mean age in months at 10‐month visit (SD)	10.18 (0.38)
Mean age in months at 16‐month visit (SD)	16.07 (0.42)
Highest maternal education level	
GCSEs or equivalent	2.3%
A Levels or equivalent	4.7%
Undergraduate degree or equivalent	36.0%
Postgraduate degree or equivalent	57.0%
Ethnicity of infant	
Asian	1.1%
Chinese	1.1%
White	85.2%
White and Asian	5.7%
White and South East Asian	3.4%
White and Latin American	2.3%
Preferred not to respond	1.1%

None of the data presented below has been previously published. Ethics approval was granted by University of Oxford Medical Sciences Interdivisional Research Ethics Committee; Ref. No. R39996/RE001 and R57972/RE001. Parents provided informed consent on behalf of their infants. At the end of each visit, families were given a BabyLab‐branded gift costing under £5. Demographic data, received from 78% of the sample, is presented in Table 1 (note that the missing demographic data is primarily from participants seen at the earliest phase of the study; we have no reason to think these participants systematically differed from those who did contribute demographic data).

### 2.2 Procedure

Tasks were completed in fixed order: Touchscreen Approach, ECITT, Touchscreen Prohibition, A‐not‐B, Toy Prohibition, alongside a broader battery of early executive function measures not included here. All tasks were completed with the infant sat in front of a table, on their parent's lap, with the experimenter sat at 90° to their right unless otherwise specified. Parents were instructed not to prompt their child during any of the tasks; any trials in which they did so were excluded from analysis. Sessions were recorded at a minimum of 30 frames per second, using two synchronised cameras positioned so that the infant's gaze and reaching response could be coded as described below.

#### 2.2.1 Touchscreen approach

Stimuli were presented on an Apple iPad tablet (screen‐size: 9.7 inches; resolution: 2048 × 1536 pixels). The experimenter drew the infant's attention to the tablet (which had previously been out of sight), on which appeared a brightly‐coloured butterfly (220 × 220 pixels) which “flew” down from the top to the centre of the screen then gently flapped its wings without changing position. The experimenter held the tablet centrally in front of the infant, pointed to the butterfly whilst saying “[Infant's name], Look, can you touch the butterfly?,” then immediately placed the tablet flat on the table centrally in front of the infant, within their reach. If touched, the butterfly made a twinkling sound and “flew” to another location on the screen, and the infant was encouraged to keep interacting with it. The following prompts were given until the butterfly was touched: After 15 s: “It's ok, you can touch it”; after 30 s: “Can you touch the butterfly?”; after 45 s: “You can touch it”; after 60 s: “Can you touch the butterfly?” If after 1 min the infant still did not tap the butterfly, the experimenter tapped it, saying “When we tap the butterfly, it moves!” and encouraged the infant to touch the butterfly for a further short period before terminating the task.

Latency to touch the butterfly after the experimenter released the tablet was coded from the video recording. Seventeen sessions were coded by a second coder and showed excellent inter‐coder reliability: ICC(single measures) = 0.997, *p *< 0.001. Infants who touched the tablet, but only after the experimenter demonstration were assigned a latency of 60 s. At the 10‐month visit, all infants touched the target before the task was terminated. Three infants at the 16‐month visit did not touch the target, even after the final encouragement, and were excluded from analysis; see Supplementary Table S1.1 for details of these exclusions and other reasons for missing data (total *n* missing = 5 at 10 months and 3 at 16 months).

#### 2.2.2 Toy prohibition

Following Friedman et al. (2011), the experimenter drew the infant's attention to an attractive toy (a glitter wand with button‐operated flashing coloured light) which had been previously out of sight. The experimenter turned the light on and held the toy out of reach centrally in front of the infant whilst saying “[Child's name], don't touch it. No,” then immediately placed the toy flat on the table centrally in front of the infant, within their reach, and released it (a small piece of adhesive material fixed to the toy prevented it rolling toward them). The experimenter then looked away from the infant and toy. If the infant had not yet touched the toy, the following prompts were given, until the toy was touched: after 30 s: “It's ok, you can touch it now”; after 35 s: “You can touch it”; after 40 s: “Let's see what happens when we touch it” (experimenter demonstrates touching the toy). If after 45 s the infant still did not touch the toy, the task was terminated.

The primary variable was latency to touch the toy. Infants who did not touch the toy until after the prohibition was released were assigned a latency of 30 s. Latency to touch was coded from the video recording. Twenty files were double coded and showed excellent inter‐coder reliability: ICC(single measures) = 1.000, *p *< 0.001. This task was added to the protocol after the study had already commenced—therefore, data is missing from a total of 28 infants at 10 months and four infants at 16 months (see Supplementary Table S1.1 for details).

#### 2.2.3 Touchscreen prohibition

Stimuli were presented on an iPad, as previously described. The experimenter drew the infant's attention to the tablet, on which appeared a brightly‐coloured frog which “hopped” down from the top to the centre of the screen. The experimenter held the tablet centrally in front of the infant, pointed to the frog whilst saying “[Child's name], don't touch it. No,” then immediately placed the tablet flat on the table centrally in front of the infant, within their reach, and released the tablet. The experimenter then looked away from the infant and tablet. If the infant tapped the frog, it made a funny sound and jumped to a different point on the screen. If the infant had not yet touched the frog after 30 s, the following prompts were given, until the frog was touched: After 30 s: “It's ok, you can touch it now”; after 35 s: “You can touch it”; after 40 s: “You can touch it” (experimenter points at the frog); after 45 s: “Let's see what happens when we touch it” (experimenter demonstrates tapping the frog). If after 1 min the infant still did not tap the frog the task was terminated and the data considered invalid (*n *= 1). Infants broke the prohibition by touching the tablet case, screen, or the frog itself; the primary variable was latency to touch any of these areas. Infants who did not touch until after the prohibition was released were assigned a latency of 30 s. Latency to touch was coded from the video recording; see Supplementary Table S1.1 for exclusions on the basis of technical and administration errors (total *n* missing = 6 at 10 months and 3 at 16 months). Seventeen files were double coded and showed excellent inter‐coder reliability: ICC(single measures) = 0.965, *p *< 0.001.

#### 2.2.4 A‐not‐B task

On a table was placed a box (46.0 × 29.7 × 12.5 cm^3^) in which were two wells (10.9 cm diameter), positioned on the same horizontal plane. The experimenter was seated across the table, facing the child. Two brown felt‐covered squares (15.5 cm^2^) were used as well covers. A trial began with the experimenter drawing the infant's attention to a toy, held centrally over the space between the wells and out of reach of the infant. When the infant was focused on the toy, the experimenter put the toy into a well, covered both wells simultaneously, then pushed the box within reach of the infant. To avoid prepotency on the ECITT being carried over to the same side in the A‐not‐B (which could inflate between‐task correlations), the initial hiding location (“A”) was counterbalanced to the ECITT such that if ECITT prepotent trials were on the left‐side of the screen, the A trial would be on the infant's right side (this decision was made prospectively, prior to any data being collected). If the infant reached to the correct well, they were praised and allowed to briefly play with the toy. If the infant reached to the incorrect well, the experimenter drew the infant's attention to the toy in the correct well and started a new trial without letting the infant touch the toy. If the infant reached to both wells simultaneously, or did not reach at all, the trial was repeated. The experimenter continued to hide the toy in the same well until the infant reached to the correct well on two consecutive trials at 10 months. To avoid ceiling effects at 16 months, inhibitory demands were increased by increasing the number of repetitions of the prepotent (“A”) trial from two to three trials. Once the criterion number of correct prepotent reaches had been achieved, the position was switched (“switch trial”) and the toy was hidden in the other well (“B”). Once the toy had been successfully retrieved from B, the position was switched again and a new round of A trials was initiated. Toys were changed regularly to maintain infants’ interest. The task continued until a total of 25 trials (33 at 16 months) had been completed, or until the infant refused to retrieve any further toys. Infants were included if they completed a minimum of 10 trials, including at least one correct reach to each location; four infants did not meet these criteria at 10 months, and two at 16 months (see Supplementary Table S1.1). A second experimenter, positioned behind the parent, out of the infant's eye‐line, recorded the infant's performance, and indicated the next hiding location. The infant's behaviour was video‐recorded and coded for accuracy and validity; see SM1. Data for 19 10‐month‐old participants and 19 16‐month‐old participants were double coded. Intercoder reliability was excellent: 10‐month accuracy *κ* = 0.98, *p *< 0.001, 10‐month validity *κ* = 0.83, *p *< 0.001, 16‐month accuracy *κ* = 0.96, *p *< 0.001, 16‐month validity *κ* = 0.89, *p *< 0.001.

Individual performance indices (calculated variables) for A‐not‐B are:

- Switching: Total number of correct switches (“A”‐to‐“B,” or “B”‐to‐“A”) as a proportion of all trials completed, such that the larger the score the better the switching ability. This score is sensitive to any short‐term prepotency built up from the reward being administered on the inhibitory (“B”) side.
- Accuracy: Proportion of A trials responded to correctly minus the proportion of B trials responded to correctly. To aid with interpretation, the Accuracy score is reversed prior to analysis (by subtracting from 1), so that a high Accuracy score corresponds to better performance. This detail was omitted from the pre‐registrations, but is the basis of our pre‐registered prediction that ECITT Accuracy would be positively associated with A‐not‐B Accuracy at 10 months.

Prior to the 16‐month pre‐registration we realised that infants with a prepotency to reach to B (e.g., due to overflow effects from the ECITT), would perform poorly on A trials and well on B trials. In these circumstances A‐not‐B Accuracy scores would not be a sensitive measure of IC. We report pre‐registered analyses using the Accuracy score at 10‐month below, for transparency, but have not included it in exploratory analyses, or pre‐registered analyses at 16 months. A‐not‐B Switching and Accuracy scores were significantly positively correlated at 10 months (*r*(76) = 0.359, *p <* 0.001) but not 16 months (*r*(72) = −0.157, *p = *0.187).

Eighty‐five 10‐month‐olds were administered both the ECITT and A‐not‐B (after which point A‐not‐B was dropped from the 10‐month protocol to allow time for piloting new measures at this time‐point once sufficient power had been achieved for the pre‐registered analyses). All 78 infants seen at 16 months were administered both the ECITT and A‐not‐B (to achieve sufficient power for the pre‐registered analyses). See Supplementary Table S1.1 for details of missing data, including exclusions (total *n* missing = 32 at 10 months and 4 at 16 months).

### 2.3 The ECITT

Stimuli were presented on an iPad, as previously described, using software and stimuli described in Holmboe et al. (2021), with the exception that the screen orientation was horizontal rather than vertical, as pilot testing indicated that 10‐month‐old infants struggled to reach the top of the screen, whereas response locations on the left and right of the screen were equally easy to reach. The experimenter held the tablet at a slight tilt, directly in front of the infant. The infant was first given at least one practice trial, allowing them to experience that tapping a centrally‐positioned blue button with a smiley‐face icon triggers a pleasing animation. In the test trials infants were shown two blue buttons, one with the smiley‐face icon, one blank. Tapping on the smiley‐face target triggered an animation, tapping on the blank button did nothing. The target appeared on the same side—henceforth “prepotent side”—in 75% of trials, and on the contralateral side—henceforth “inhibitory side”—in 25% of trials. Each trial sequence consisted of 32 trials in random order, with the constraints that for the first three trials the target was on the prepotent side, and that there were never more than two inhibitory trials and five prepotent trials in a row. In six cases (10‐month session 1 *n *= 3, 16‐month *n *= 3), infants had to restart the task after a technical issue. To accommodate potential disruption to data representativeness, the number of trials considered was increased to 40 for those infants. To ensure that a prepotency was established as intended, if on the first test trial the infant's initial response was to tap the inhibitory side, the prepotent and inhibitory locations were reversed. The prepotent and inhibitory locations were reversed between the 10‐ and 16‐month visits, but were consistent between the first and second 10‐month visits.

The tablet recorded the accuracy and reaction time (RT; in milliseconds) of each response. These were checked and corrected (where necessary) via coding from video recordings; see Supplementary Material (SM1) for details. Accuracy and validity checks were double‐coded for 17 10‐month‐olds and 30 16‐month‐olds. Inter‐coder reliability was excellent: 10‐month accuracy *κ* = 0.97, *p *< 0.001, 10‐month validity *κ* = 0.88, *p *< 0.001, 16‐month accuracy *κ* = 0.94, *p *< 0.001, 16‐month validity *κ* = 0.85, *p *< 0.001, RT corrections (on an additional set of 20 participants) *r *= 0.974, *p *< 0.001.

RT data were only analysed at 16 months because responses at 10 months were often characterised by turning intermittently away from the tablet, and/or trying to grab or mouth the tablet. These behaviours meant individual RTs were likely not an accurate index of inhibitory performance at this age.

Individual performance indices (calculated variables) for the ECITT are:

- Prepotent Accuracy (PAcc): Proportion of correct responses out of all valid prepotent trials.
- Inhibitory Accuracy (IAcc): Proportion of correct responses out of all valid inhibitory trials.
- Accuracy Difference (AccD): PAcc minus IAcc. To aid with interpretation, the AccD score is reversed prior to analysis (by subtracting from 1), so that a high AccD score corresponds to better performance. This detail was omitted from the pre‐registrations, but is the basis of our pre‐registered prediction that ECITT Accuracy would be positively associated with A‐not‐B Switching at 16 months.
- Switching score: The number of correct switches made from prepotent location to inhibitory location and vice versa, as a proportion of all trials completed, such that the larger the score the better the switching ability. This score is sensitive to any short‐term prepotency built up from the inhibitory side being rewarded. Consistent with pre‐registered expectations, ECITT Switching and AccD scores were positively correlated at 10 months (*r*(84) = 0.412, *p <* 0.001) and 16 months (*r*(68) = 0.531, *p <* 0.001).
- Inhibitory RT (16 months only): The median RT for all correct and valid inhibitory trials
- Prepotent RT (16 months only): The median RT for all correct and valid prepotent trials.

As per the pre‐registration, participants with PAcc lower than 60% were excluded from analyses (*n *= 7 at 10 months and *n *= 6 at 16 months) as less than 60% is considered an indication of random performance, meaning that no prepotent response had been built up (Holmboe et al., 2021). Data were also excluded if fewer than 10 valid trials and/or fewer than two inhibitory trials were completed, as per the pre‐registration (*n *= 12 at 10 months and *n *= 1 at 16 months). See Supplementary Table S1.1 for details of these exclusions and other reasons for missing data (total *n* missing = 24 at 10 months and 9 at 16 months).

### 2.4 Data analysis plan

Sex differences were tested for using independent samples *t*‐tests (Mann‐Whitney U for non‐normally‐distributed Touchscreen Approach, prohibition and A‐not‐B switching variables). Condition effects in the competitive inhibition tasks (the ECITT and A‐not‐B) were tested for using paired sample *t*‐tests. Test‐retest associations, within‐construct, across‐construct and longitudinal associations were tested for using correlational tests (Spearman's *rho* used for skewed Touchscreen Approach, prohibition and A‐not‐B switching variables, and Pearson's for all other variables). Developmental progression was tested for using paired sample *t*‐tests (Wilcoxon Signed Ranks Test for skewed Touchscreen Approach and prohibition variables). Pre‐registered tests were conducted using one‐tailed tests, with an alpha of 0.05, all other tests were two‐tailed, with an alpha of 0.05. The false discovery rate was corrected for using the Benjamini‐Hochberg correction for each set of family‐wise tests. In supplementary analyses (see Supplementary Materials 3), we used multiple imputation to account for data loss due to technical or protocol reasons: all conclusions remain unaffected.

## 3 RESULTS

**FIGURE 1:**
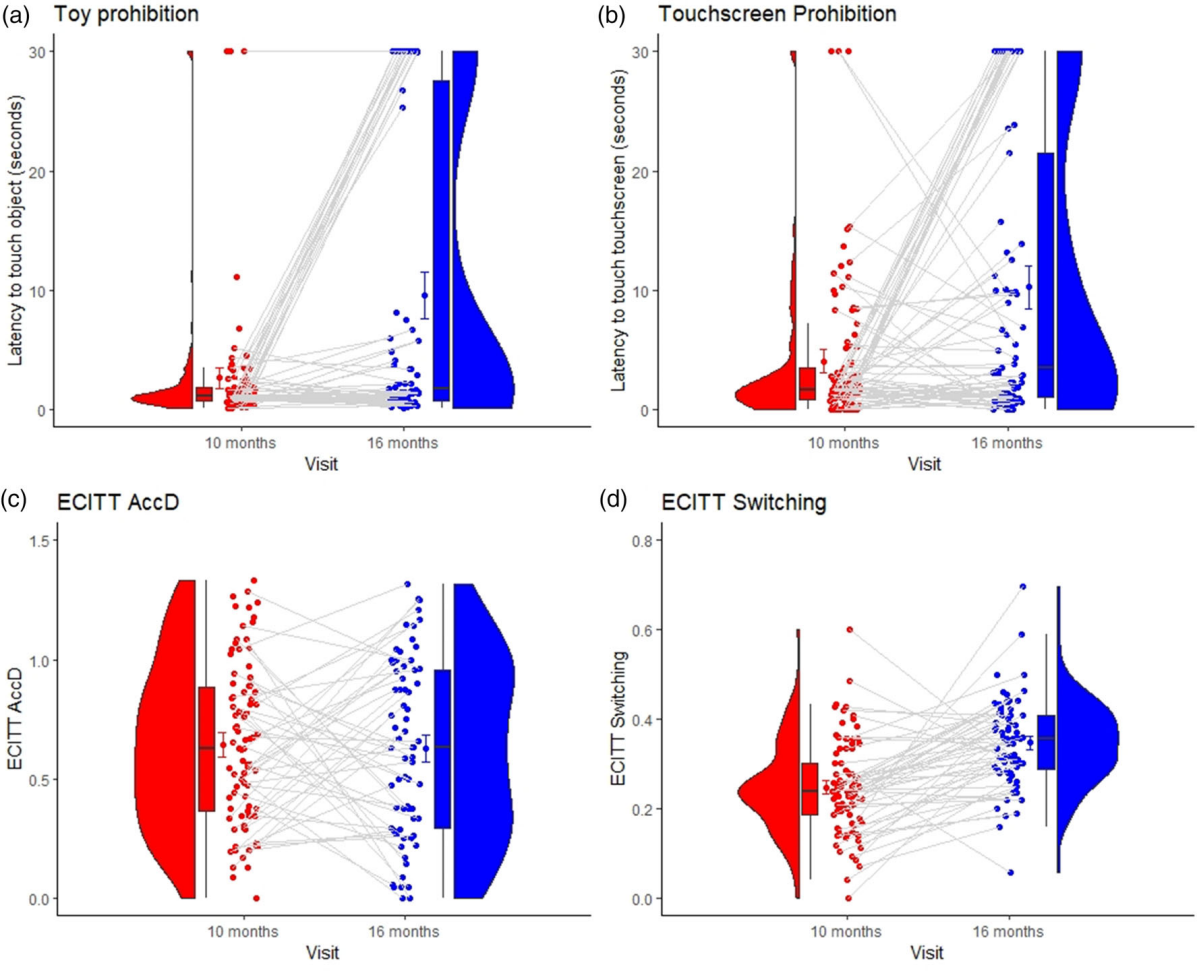
Repeated measures plots for: Toy Prohibition (a) Touchscreen Prohibition (b), ECITT AccD (c), and ECITT switching (d). ECITT: Early Childhood Inhibitory Touchscreen Task; AccD: Accuracy Difference Plot elements shown (from left) are: distribution density; box plot indicating the interquartile range (box upper and lower bounds) and median (horizontal band); mean (coloured dot) with 95% confidence interval (t‐bar whiskers); individual observations; and individual repeated measures regression lines

Descriptive data for task performance at 10 and 16 months are shown in Table 2 and Figure 1. As shown in Supplementary Table S2.1, no sex differences were observed for any of the tasks. Therefore, sex is not included as a covariate in any subsequent analyses and is not considered further.

**TABLE 2:** Task performance

Construct	Variable			10 months	16 months
Behavioural Inhibition	Touchscreen approach (latency in seconds)		Mean	12.786	17.434
			SD	15.611	22.399
			Min, Max	0.00, 60.00	00, 60.00
			Median	5.810	5.675
Directed global inhibition	Touchscreen Prohibition (latency in seconds)		Mean	4.046	10.075
			SD	6.391	11.701
			Min, Max	0.00, 30.00	0.00, 30.00
			Median	1.620	3.440
	Toy Prohibition (latency in seconds)		Mean	2.617	9.296
			SD	5.678	12.564
			Min, Max	0.10, 30.00	0.10, 30.00
			Median	1.069	1.651
Competitive inhibition	Early Childhood Inhibitory Touchscreen Task (ECITT)	Accuracy Difference (AccD)	Mean	0.642	0.619
			SD	0.341	0.368
			Min, Max	0.000, 1.333	0.000, 1.318
			Median	0.624	0.628
		Switching	Mean	0.246	0.347
			SD	0.101	0.102
			Min, Max	0.000, 0.600	0.056, 0.696
			Median	0.238	0.356
			Min, Max	0.000, 1.000	0.053, 1.000
	A‐not‐B task	A‐not‐B accuracy	Mean	0.720	0.884
			SD	0.426	0.380
			Min, Max	0.000, 1.565	0.095, 1.906
			Median	0.742	0.927
		A‐not‐B switching	Mean	0.187	0.179
			SD	0.164	0.115
			Min, Max	0.000, 0.680	0.000, 0.438
			Median	0.139	0.182

Abbreviation: SD, standard deviation.

### 3.1 Is the Touchscreen Prohibition task suitable as a measure of directed global inhibition in infancy?

Individual differences in performance on Touchscreen Prohibition and the Toy Prohibition task showed a moderate positive correlation (*r_s _* = 0.347) at 10 months (see Table 3), and a strong positive correlation (*r_s _* = 0.591) at 16 months (see Table 4). At 10 months, there was no significant association between Touchscreen Approach and Toy Prohibition, but there was a significant association between Touchscreen Approach and Touchscreen Prohibition performance (*r_s _* = 0.308) (see Table 3). To investigate this further we ran an exploratory multiple linear regression of 10‐month Touchscreen Prohibition performance on 10‐month Touchscreen Approach and Toy Prohibition performance (*F*(2,76) = 16.44, *p *< 0.001, *R* ^2 ^= 0.302). In this model, 10‐month Touchscreen Prohibition performance was significantly predicted by Toy Prohibition performance (*β *= 0.522, *p *< 0.001) but not by Touchscreen Approach performance (*β *= 0.136, *p *= 0.160). At 16 months, Touchscreen Approach showed no significant association with either Touchscreen Prohibition or Toy Prohibition (see Table 4). In combination, these results confirm our hypothesis that the Touchscreen Prohibition task elicits variation that can reasonably be attributed to directed global inhibition, particularly at 16 months.

**TABLE 3:** Bivariate associations between tasks at 10 months

	Touchscreen prohibition (DGI)	Toy prohibition (DGI)	A‐not‐B switching (CI)	ECITT AccD (CI)	ECITT Switching (CI)
Touchscreen approach (BI)	0.308^a^ [0.106, 0.484] *102*	0.230 [−0.006, 0.443] *80*	−0.044 [−0.275, 0.186] *76*	0.057 [−0.159, 0.283] *84*	0.099 [−0.110, 0.299] *91*
Touchscreen prohibition (DGI)		0.347^a^ [0.122, 0.553] *79*	−0.180 [−0.413, 0.070] *76*	0.193 [−0.037, 0.403] *84*	0.134 [−0.073, 0.347] 90
Toy prohibition (DGI)			−0.202 [−0.438, 0.058] *56*	0.067 [−0.180, 0.306] *70*	0.086 [−0.144, 0.317] *74*
A‐not‐B Accuracy (CI)				0.047 [−0.175, 0.291] *63*	0.032 [−0.298, 0.298] 68
A‐not‐B switching (CI)				0.191 (0.177)^b^ [−0.064, 0.440] *63*	0.093 (0.054)^b^ [−0.189, 0.333] *68*

*Note*: Cells show the correlation coefficient, 95% Confidence Interval based on 1000 bootstrap samples in square brackets, and n in italics. Variable names indicate the target construct in parentheses.

Abbreviations: BI, behavioural inhibition; DGI, directed global inhibition; CI, competitive inhibition; ECITT, Early Childhood Inhibitory Touchscreen Task; AccD, accuracy difference.

^a^ Significant after applying the Benjamini‐Hochberg correction for 16 tests with an alpha of 0.05.

^b^ A‐not‐B switching correlations presented for Pearson's with Spearman's *rho* in brackets, due to high skew.

Exploratory tests use Spearman's *rho* used for analyses involving skewed Touchscreen Approach and both Prohibition variables, and Pearson's for all other correlations.

Pre‐registered tests (underlined) are one‐tailed, all other tests two‐tailed.

**TABLE 4:** Bivariate associations between tasks at 16 months

	Touchscreen prohibition (DGI)	Toy prohibition(DGI)	A‐not‐B switching (CI)	ECITT AccD (CI)	ECITT Switching (CI)
Touchscreen approach (BI)	0.125 [−0.135, 0.374] *74*	−0.057 [−0.294, 0.158] *71*	0.113 [−0.155, 0.355] *71*	0.207 [−0.026, 0.431] *66*	−0.020 [−0.264, 0.205] *66*
Touchscreen prohibition (DGI)		0.591^a^ [0.376, 0.753] *72*	0.068 [−0.185, 0.310] *72*	0.157 [−0.090, 0.383] *66*	−0.114 [−0.347, 0.123] *66*
Toy prohibition (DGI)			0.002 [−0.240, 0.247] *73*	0.092 [−0.170, 0.342] *64*	0.024 [−0.195, 0.265] *64*
A‐not‐B switching (CI)				0.447 ^a^ (0.437) ^b^ [0.216, 0.638] *64*	0.313 ^a^ (0.165) ^b^ [0.011, 0.532] *64*

*Note*: Cells show the correlation coefficient, 95% Confidence Interval based on 1000 bootstrap samples in square brackets, and n in italics. Variable names indicate the target construct in parentheses.

Abbreviations: BI, behavioural inhibition; DGI, directed global inhibition; CI, competitive inhibition; ECITT, Early Childhood Inhibitory Touchscreen Task; AccD, accuracy difference.

^a^ Significant after applying the Benjamini‐Hochberg correction for 14 tests with an alpha of 0.05.

^b^ A‐not‐B switching correlations presented for Pearson's with Spearman's *rho* in brackets, due to high skew.

Exploratory tests use Spearman's *rho* used for analyses involving skewed Touchscreen Approach and both Prohibition variables variables, and Pearson's for all other correlations. Pre‐registered tests (underlined) are 1‐tailed, all other tests 2‐tailed.

### 3.2 Is the ECITT suitable as a measure of competitive inhibition in infancy?

#### 3.2.1 Evidence for condition effects on the ECITT and A‐not‐B task

As shown in Table 5, infants were more accurate and faster on ECITT prepotent trials compared with inhibitory trials—with large effect sizes for accuracy scores, and a small effect for 16‐month RT. These results indicate that the ECITT elicits condition effects that can reasonably be attributed to competitive inhibition.

**TABLE 5:** Performance on the ECITT at 10 and 16 months

	Inhibitory accuracy	Prepotent accuracy	Proportion correct on inhibitory versus prepotent trials		
	Mean (SD)	Mean (SD)	Test statistic	*p*	Effect size (d)
**10 months Session 1**	0.494 (0.292)	0.852 (0.118)	*t*(83) = 9.616	<0.001	1.049
**10 months Session 2**	0.530 (0.269)	0.856 (0.116)	*t*(51) = 8.197	<0.001	1.137
**16 months**	0.518 (0.336)	0.896 (0.108)	*t*(67) = 8.544	<0.001	1.036

	**Inhibitory RT**	**Prepotent RT**	**RT on correct inhibitory versus prepotent trials**		
	Mean (SD)	Mean (SD)	Test statistic	*p*	Effect size (d)
**16 months**	1703 (506)	1518 (354)	*t*(57) = −2.820	0.007	0.370

Abbreviation: RT, reaction time.

At both timepoints, infants had poorer ECITT AccD scores when the prepotent side was on the right, but ECITT switching scores were not significantly affected by prepotent side (see SM 4.1).

In comparison, on the A‐not‐B task, although infants were more accurate on prepotent (“A”) trials compared with inhibitory (“B”) trials the effect size was moderate at 10 months and small at 16 months; see Table 6. There was no evidence for spill‐over effects from ECITT to A‐not‐B (see SM 4.2).

**TABLE 6:** Performance on the A‐not‐B task at 10 and 16 months

	Proportion “B” trials correct	Proportion “A” trials correct	Proportion correct on A versus B trials		
	Mean (SD)	Mean (SD)	Test statistic	*p*	Effect size (d)
**10 months Session 1**	0.435 (0.315)	0.706 (0.251)	*t*(75) = 5.714	<0.001	0.655
**16 months**	0.613 (0.262)	0.717 (0.240)	*t*(71) = 2.592	0.012	0.305

#### 3.2.2 ECITT test‐retest reliability

Pre‐registered one‐tailed Pearson's correlations indicated a positive association between ECITT AccD scores (*r *= 0.303, *p *= 0.018 [CI: 0.032, 0.514]), and between ECITT Switching Scores (*r *= 0.602, *p *< 0.001 [CI:0.387, 0.767]) at the test and re‐test visits (see SM4.3 for IAcc and PAcc test‐retest reliability).

#### 3.2.3 Associations between performance on the ECITT and A‐not‐B task

As hypothesized, ECITT AccD scores and ECITT switching scores were positively associated with A‐not‐B switching scores at 16 months (*r *= 0.447 and *r *= 0.313; see Table 5). The hypothesized positive association between ECITT switching scores and A‐not‐B switching scores at 10 months did not meet significance thresholds, nor was there a significant association between ECITT AccD scores and either A‐not‐B performance score at 10 months (see Table 4).

### 3.3 Do infants show developmental progression in terms of directed global inhibition and competitive inhibition between the ages of 10 and 16 months?

As hypothesized, 16‐month‐olds were able to delay touching a prohibited target for longer than 10‐month‐olds for both Touchscreen Prohibition (*Z* = −2.911, *p *= 0.003) and Toy Prohibition (*Z *= −2.845, *p *= 0.004) tasks (see Table 7 and Figure 1).

**TABLE 7:** Performance changes from 10 to 16 months on the behavioural inhibition, global directed inhibition, and competitive inhibition tasks

Construct	Variable	Developmental progression		Longitudinal stability		
		Test statistic	*p*	Correlation coefficient	Confidence interval	n
**Behavioural inhibition**	Touchscreen approach	*Z *= −0.648	0.520	−0.137	−0.392, 0.143	66
**Directed global inhibition**	Touchscreen prohibition	*Z *= −2.911	0.003	0.037	−0.185, 0.268	*66*
	Toy prohibition	*Z *= −2.845	0.004	0.027	−0.251, 0.315	*52*
**Competitive inhibition**	ECITT AccD	*t *= −0.120	0.905	0.034	−0.240, 0.304	*47*
	ECITT switching	*Z *= −5.164	<0.001	0.125	−0.099, 0.366	*50*
	A‐not‐B Switching	*NA*		0.024 (0.029) ^a^	−0.251, 0.275	*44*

Abbreviations: ECITT, Early Childhood Inhibitory Touchscreen Task; AccD, accuracy difference.

^a^ A‐not‐B correlations presented for Pearson's with Spearman's *rho* in brackets, due to high skew. Spearman's *rho* used for analyses involving skewed Touchscreen Approach and both Prohibition variables, and Pearson's for ECITT AccD correlations. Pre‐registered tests (underlined) are one‐tailed, all other tests two‐tailed.

As hypothesized, ECITT switching scores significantly increased between 10 and 16 months (*Z *= −5.164, *p *< 0.001). However, ECITT AccD scores were not significantly higher at 16 months compared with 10 months (see also Table 7 and Figure 1) (note that progression on A‐not‐B was not tested due to differences in the protocol between the time‐points).

### 3.4 Do individual differences in performance on inhibitory control and behavioural inhibition measures show longitudinal stability between the ages of 10 and 16 months?

Contrary to our hypotheses, there was little evidence for longitudinal stability on either prohibition task (see Table 7).

Our hypothesis that scores on the ECITT at 10 months would be positively correlated with ECITT scores using the same variable at 16 months was not supported by the primary analyses (see Table 7). However, there was a moderate association between 10‐month and 16‐month ECITT switching scores after excluding extreme values in pre‐registered supplementary analyses (see Supplementary Table S4.4.4).

Contrary to our hypothesis, there was no evidence for longitudinal stability on the Touchscreen Approach task (see Table 7).

### 3.5 Is there evidence for associations between different components of IC, and between measures of IC and behavioural inhibition?

Contrary to our hypotheses, there were no significant cross‐sectional associations between latency to touch on the Touchscreen Approach task and any of the ECITT or A‐not‐B variables at either 10 or 16 months (see Tables 3 and 4).

Exploratory analyses revealed no significant cross‐sectional or longitudinal associations between latency to touch on either of the prohibition tasks with either ECITT or A‐not‐B performance (see Tables 3, 4, and 8).

**TABLE 8:** Bivariate correlations between behavioural inhibition and inhibitory control (IC) measures at 10‐ and 16‐months

	16 m Touchscreen approach (BI)	16 m Touchscreen prohibition (DGI)	16 m Toy prohibition (DGI)	16 m ECITT AccD (CI)	16 m ECITT Switching (CI)	16 m A‐not‐B switching (CI)
10 m Touchscreen approach (BI)	*–*	−0.109 [−0.349, 0.148] *67*	−0.145 [−0.387, 0.123] *67*	0.235 [−0.032, 0.483] *59*	0.277 [0.010, 0.509] *59*	−0.038 [−0.309, 0.191] *66*
10 m Touchscreen prohibition (DGI)	−0.015 [−0.273, 0.238] *65*	–	0.099 [−0.128, 0.344] *66*	0.083 [−0.196, 0.359] *58*	0.220 [−0.090, 0.493] *58*	−0.001 [−0.284, 0.274] *65*
10 m Toy prohibition (DGI)	−0.184 [−0.442, 0.083] *53*	−0.033 [−0.297, 0.242] *53*	*–*	0.255 [−0.072, 0.524] *46*	0.325 [−0.073, 0.511] *46*	0.070 [−203, 0.330] *51*
10 m ECITT AccD (CI)	−0.124 [−0.417, 0.182] *53*	0.251 [−0.023, 0.504] *54*	0.151 [−0.104, 0.415] *54*	–	*–*	–
10 m ECITT switching (CI)	−0.025 [−0.288, 0.236] *56*	0.064 [−0.187, 0.315] *57*	0.025 [−0.249, 0.320] *57*	–	*–*	−0.175 (−0.224) [427, 0.081] *56*
10 m A‐not‐B switching (CI)	0.337 [0.035, 0.602] *44*	−0.070 [−0.359, 0.221] *45*	−0.183 [−0.450, 0.112] *45*	−0.032 (−0.016) [−0.333, 0.278] *41*	0.125 (0.131) [−0.204, 0.402] *41*	–

*Note*: Cells show the correlation coefficient, 95% Confidence Interval in square brackets (based on 1000 bootstrap samples) and n in italics. Variable names indicate the target construct in parentheses. A‐not‐B and ECITT switching correlations presented for Pearson's with Spearman's rho in brackets, due to high skew. Spearman's rho used for analyses involving skewed Touchscreen Approach and both Prohibition variables, and Pearson's for ECITT AccD correlations. Pre‐registered tests (underlined) are two‐tailed, all other tests two‐tailed.

Abbreviations: BI, behavioural inhibition; DGI, directed global inhibition; CI, competitive inhibition; M, month; ECITT, Early Childhood Inhibitory Touchscreen Task; AccD, accuracy difference.

Conclusions were unaffected by excluding extreme scores (SM 4.4), or inhibitory trials which immediately followed another inhibitory trial (SM 4.5).

## 4 DISCUSSION

In this study, we measured infants’ performance on a range of IC tasks, at ages 10 and 16 months. We used a novel (Touchscreen Prohibition) and an established (Toy Prohibition) measure of directed global IC, a novel (the ECITT), and an established (A‐not‐B task) measure of competitive IC, and a novel measure of behavioural inhibition (Touchscreen Approach). We demonstrate that our novel measures of IC are suitable for use with this age group, but that within‐construct coherence of performance across tasks is higher in toddlerhood compared with infancy. Our data show evidence of developmental progression in directed global inhibition, and in some aspects of competitive inhibition, but not of longitudinal stability of individual differences, nor of developmental associations between directed global inhibition, competitive inhibition and behavioural inhibition. Below, we expand on these results, and reflect on their implications for theory and practice in the context of this Special Issue by detailing the strengths and limitations of our two new measures for assessment of IC in infancy, and reflecting on what our results mean for theoretical models of development of this important aspect of executive function.

### 4.1 Two new measures of early inhibitory control

The first aim of this study was to broaden the repertoire of IC tasks that are available to infancy researchers by presenting a battery of short, engaging tasks, suitable for both infants and toddlers, that can be used to target directed global inhibition and competitive inhibition.

#### 4.1.1 Suitability of the Touchscreen Prohibition task as a measure of directed global inhibition

In terms of directed global inhibition, associations between performance on our first novel task—the Touchscreen Prohibition task—with another commonly‐used infant prohibition task—the Toy Prohibition task—were moderate at 10 months, and strong at 16 months. Further, although performance on the Touchscreen Prohibition task was also significantly associated with latency to touch on our behavioural inhibition task at 10 months, exploratory multiple regression analysis indicated that variation in performance on the Touchscreen Prohibition task was better predicted by Toy Prohibition performance than by behavioural inhibition. We, therefore, conclude the Touchscreen Prohibition task to be an appropriate measure of directed global inhibition for this age group.

#### 4.1.2 Suitability of the ECITT as a measure of competitive inhibition

In terms of competitive inhibition, our results demonstrate that, as intended, our second novel task, the ECITT, elicits competitive inhibition effects as early as 10 months of age. At both 10‐ and 16‐months, infants were less accurate on trials where the correct response location was infrequent (“inhibitory trials”) compared with trials where the correct response location was well‐primed (“prepotent trials”). Moreover, 16‐month‐olds were slower on correct inhibitory trials compared with correct prepotent trials. ECITT scores in general, and switching scores (accuracy on trials where the correct location was contralateral to the previously‐rewarded location) in particular, showed satisfactory test‐retest reliability for the purposes of evaluation of early IC ability across a 1‐week interval. Further evidence for construct validity of the ECITT as a measure of competitive inhibition is provided by the moderate positive association observed between performance on the ECITT and a more established measure of competitive inhibition—the A‐not‐B task—at 16 months, consistent with pre‐registered hypotheses.

However, contrary to predictions, we did not find a significant association between ECITT and A‐not‐B performance at 10 months. One possibility is that processes other than competitive inhibition influence ECITT and/or A‐not‐B performance in infancy to a varying extent. For example: using impoverished visual input (identical lids) in A‐not‐B may provide less cues to enable infants to overcome the prepotent response tendency built up during the task compared with the ECITT (where the correct location is cued with a smiley face) (Thelen et al., 2001); there is greater potential for pragmatic misinterpretation in A‐not‐B compared to the ECITT due to higher researcher involvement in the hiding event (Topál et al., 2008); and the differing motor control demands of reaching in the tasks (in A‐not‐B the targets were both further apart from each other, and from the infant, compared to in the ECITT) may impact performance (Gottwald et al., 2016; Thelen et al., 2001). Individual differences in each of these processes can be expected to be more pronounced in infancy, whilst these skills are still emergent, compared with toddlerhood. Alternatively, or additionally, it may be the case that the neural mechanisms underlying competitive inhibition are still in transition in infancy. We discuss this possibility further with regards to our findings on longitudinal stability.

### 4.2 Insights into the development of inhibitory control

#### 4.2.1 Progression and longitudinal (in)stability in the development of directed global inhibition

The second aim of this study was to provide new insights into developmental progression, longitudinal stability and cross‐component associations involved in the early development of IC, as a first step towards establishing the plausibility of competing models of early IC development. Evidence of developmental progression between 10 and 16 months in directed global inhibition was clear from performance on both prohibition tasks and was broadly consistent with improvements reported in other studies (Frick et al., 2019; Friedman et al., 2011). Counter to our hypothesis however, there was no evidence for longitudinal stability between 10 and 16 months on either prohibition task.

The lack of stability in individual differences on directed global inhibition in this study could be attributable to transitions in frontoparietal networks between the ages of 10 and 16 months, as discussed in more detail in the section below. However, we may also have been unable to detect stability in individual differences due to floor effects at 10 months (see Figure 1). Consistent with our findings, Friedman et al. (2011) found that even infants characterised as showing high restraint based on their longitudinal Toy Prohibition performance (between 14 and 36 months) had a 64% likelihood of touching the toy within 10 s of the prohibition being issued at the first assessment point at 14 months of age. This indicates, as with our data, that “poor” prohibition performance is normative in infancy. Below we examine two possible interpretations of this poor performance.

The most obvious account is that at age 10 months, infants’ directed global inhibition skills tend to be so weak that, as a group, they are unable to resist reaching to the prohibited object. The pattern of data shown in Figure 1b is somewhat compatible with this interpretation in that it appears that a reasonable proportion of the sample initially hesitated before “giving in” and reaching to tap the touchscreen. This behavioural profile seems less‐common on the Toy prohibition task Figure 1a, perhaps because the salience of a 3D object is stronger for this age group such that greater levels of directed global inhibition skills are required to even hesitate in reaching to the toy. An alternative interpretation of our data is that non‐compliance with the prohibition (i.e., reaching to touch the prohibited object) is attributable to some other factor than directed global inhibition. Although the language demands of the tasks were kept as low as possible, the prohibition was delivered verbally (“No, don't touch”), albeit with accompanying tone and facial expression cues, such that it is possible that at 10 months some infants lacked the language comprehension skills to understand the task requirements. Additionally or alternatively, the unusual circumstance of having a novel, engaging object placed within reach by the same person who then says not to touch, might be sufficient to induce pragmatic misinterpretation of the task. Put simply: infants are confused about the task requirements and so default to their preferred behaviour (reaching for the object). To rule out this option, we recommend that parent report of infants’ responses to prohibition in day‐to‐day, ecologically‐valid, contexts should be used alongside performance measures in future studies.

#### 4.2.2 Progression and (in)stability in the development of competitive inhibition

As with directed global inhibition, our results provide some evidence for developmental improvement in competitive inhibition between 10 and 16 months, in that ECITT switching performance scores increased between the two time‐points. These findings extend the evidence base for increases in competitive inhibition skills with age, which has previously been demonstrated in toddlers and pre‐schoolers (Holmboe et al., 2021; Petersen et al., 2016). However, this improvement with age was not observed for the ECITT AccD variable: this inconsistency may yield important insights into the nature of prepotency and IC in early development. Specifically, we propose that our data indicate the influence of two types of prepotency on inhibitory performance in infancy and toddlerhood; an immediate (i.e., trial‐to‐trial) prepotency—indexed by the switching variable—and a slower probability‐based prepotency influenced by the cumulative likelihood of a target being rewarded or reached towards—indexed by the accuracy difference score. Our results therefore may indicate that toddlers are on their way towards responding flexibly in terms of being more able, compared with when they were infants, to override immediate prepotencies (switching scores improve between 10 and 16 months). However, exerting IC over probability‐based prepotencies appears to be just as challenging for toddlers as infants (16‐month‐olds showed no advantage on ECITT accuracy difference scores over 10‐month‐olds).

Our results also suggest that probability‐based prepotencies are influenced by existing biases. Infants have been found to demonstrate, on average, a bias towards right‐handed reaching from as early as 6 months of age (Ferre et al., 2010), as well as a preference for reaching to objects positioned on the ipsilateral side to their reaching hand, which pervades into toddlerhood (Begum Ali et al., 2020). In our study, ECITT accuracy difference scores (but not switching scores) at both 10 and 16 months were poorer when the well‐primed side was on the right, indicating that prepotencies may be enhanced by existing right‐hand biases. An interesting avenue for future research is to test whether the influence of prior side biases on probability‐based prepotencies reduces over the course of a task with increasing repetitions of the dominant location.

In our data, contrary to predictions, we found no evidence of longitudinal stability on the A‐not‐B switching measure between 10 and 16 months. For ECITT, weak‐to‐moderate associations were observed for the switching measure only, and only when several extreme values were removed. Longitudinal stability in competitive inhibition between infancy and toddlerhood therefore appears to be low or, at best, inconsistent. Research on the neural basis for competitive inhibition in infancy is sparse, but amongst older children and adults, this skill is considered to rely heavily on the prefrontal cortex (PFC) (Chambers et al., 2009; Fiske & Holmboe, 2019). Post‐mortem tissue analysis provides evidence for a transitional period in the structure and organisation of PFC at around 16 months (Petanjek et al., 2008; Sedmak et al., 2018). Further, in vivo brain imaging indicates that frontoparietal networks show non‐adult‐like topologies at age 1 year, but by 2 years show a more adult‐like organisation (Fan et al., 2011; Gao et al., 2014). Such transitions may be implicated in the experience‐dependent fine‐tuning of brain functional organization, considered to be of particular importance to executive functions (Johnson, 2011; Karmiloff‐Smith, 1995, 2010). Variability in early experience, and transitions in neural network re‐organisation, may well impact on individual performance differences, including shifts in who performs well in infancy versus toddlerhood.

### 4.3 Associations between directed global inhibition, competitive inhibition and behavioural inhibition

We did not find evidence to support the argument that the slow speed of approach characteristic of behavioural inhibition may support the development of voluntary, effortful inhibition (i.e., directed global and competitive inhibition), as suggested by Aksan and Kochanska (2004). Contrary to our hypotheses, in our data, latency to touch on a behavioural inhibition task (Touchscreen Approach) was not significantly correlated with performance on either competitive inhibition task (Tables 3 and 4). Nor did our findings support a model in which stronger directed global inhibition facilitates higher performance scores on competitive inhibition scores via the reduction of impulsive responses. Results of exploratory cross‐sectional correlational tests (also Tables 3 and 4) indicated no significant associations between our prohibition measures and of our competitive inhibition measures (the A‐not‐B task and the ECITT). Further, our data do not support a hierarchical model of IC development in which directed global inhibition supports the *development* of competitive inhibition: results of exploratory longitudinal correlational tests (Table 8) indicated no significant associations between performance on our prohibition measures at 10 months and our competitive inhibition measures (the A‐not‐B task and the ECITT) at 16 months. Thus, our findings indicate instead that competitive inhibition and directed global inhibition skills initially follow distinct developmental pathways in infancy and that IC should not be considered a stable, uni‐dimensional construct during infancy.

These results should be considered in the light of three caveats. Firstly, the prohibition task floor effects at 10 months previously discussed may have limited the possibility of detecting longitudinal associations with competitive inhibition measures; follow‐up research is therefore merited to test whether such associations emerge in toddlerhood, once directed global inhibition skills have become more established. Secondly, due to testing constraints, after exclusions, the sample for longitudinal correlations ranged from 41 to 67, providing 63%–82% power to detect an effect size of 0.3 with our planned one‐tailed tests, and 50%–72% power to detect an effect size of 0.3 with exploratory tests (64%–72% after imputation for cross‐sectional missing data, as outlined in SM3). It is possible therefore that genuine weak associations between variables went undetected due to lack of power to detect them, and thus future studies should consider recruiting larger samples to rule out this possibility.

A further consideration is that it is possible, particularly at the 10‐month‐timepoint, that variation in motor control may also have contributed to variation in reaching response speeds and thus potentially, via that pathway, to variation in pre‐potency strength on the ECITT and A‐not‐B (Thelen et al., 1996). This additional source of variation may have masked genuine associations with directed global inhibition or behavioural inhibition. Further, as 16‐month‐olds are likely to be generally capable of faster and more‐precise motor responses compared with 10‐month‐olds, pre‐potencies may have had less time to dissipate at 16 months, and thus the competitive inhibition demands of the tasks may have been higher at 16 months compared with 10 months. These increased demands could account for why developmental progression was not observed on ECITT task performance between 10 and 16 months. To explore this possibility, future studies could include eye‐tracking measures of competitive inhibition alongside reaching‐based tasks such as the ECITT and A‐not‐B, to facilitate the partialling out of motor control from competitive inhibition performance.

### 4.4 Implications for research methods

The methodological implication of our conclusion that IC should not be considered a stable, uni‐dimensional construct during infancy is that researchers interested in measuring IC in infancy should avoid using a single measure as a proxy for all types of IC. Further, the modest correlations found even between tasks designed to capture the same construct indicate the need to take steps to minimise the effects of measurement impurity. It is beyond the scope of this study to define what such steps should be in terms of analytic approach—see instead Camerota et al. (2020) for useful discussion—but in terms of measurement selection we recommend, on the basis of these findings, to include at least two performance‐based indicators of directed global and competitive inhibition respectively. We have outlined above the strengths and weaknesses of four specific tasks that may be used as such indicators, but also note the potential utility of including a parent report measure of IC to mitigate the ecological validity issues previously mentioned.

### 4.5 Implications for applied research

A commonly‐stated objective in developmental research is to lower the age‐boundary at which early difficulties in important cognitive functions—such as IC—can be detected, in order to affect positive change through intervention before such difficulties become entrenched or implicated in other down‐stream difficulties (Anderson & Reidy, 2012; Hendry et al., 2016). On the basis of the data presented in this study, which showed low longitudinal stability on all but one of our markers of directed global and competitive inhibition (and even then, only when extreme values were excluded) we advise caution in attempting to screen for difficulties based on performance data alone within the first year of life. This is not to say that variation in IC in infancy is meaningless; rather that, during this period of rapid change in multiple inter‐related skills, other indicators of risk and resilience (e.g., gestational, genetic, or environmental) and phenotypic variation (e.g., neural markers) may be more informative than behavioural performance alone (Johnson et al., 2021).

## 5 CONCLUSIONS

Our results showcase the complexity of IC development and demonstrate that infants show variation in competitive inhibition, directed global inhibition, and behavioural inhibition from as early as 10 months of age. Performance between measures targeting similar aspects of IC show increasing coherence, and some developmental progression, in the transition from infancy to toddlerhood. However, early task‐specific strengths do not appear to carry forward into the second year of life. The weak cross‐sectional and longitudinal associations observed between constructs indicate that researchers interested in measuring IC in infancy should consider performance indicators of both directed global inhibition and of competitive inhibition, such as those presented in this paper.

## CONFLICT OF INTEREST

The authors report no conflicts of interest.

## CREDIT AUTHOR STATEMENT

Alexandra Hendry: Conceptualisation, Methodology, Formal Analysis, Investigation, Writing – Original Draft, Writing – Review and Editing, Visualisation, Validation. Isobel Greenhalgh: Investigation. Rhiannon Bailey: Formal Analysis. Abigail Fiske: Methodology, Formal Analysis. Henrik Dvergsdal: Software, Methodology, Data Curation. Karla Holmboe: Conceptualisation, Methodology, Investigation, Resources, Data Curation, Writing – Review and Editing, Funding Acquisition, Project Administration, Supervision.

## Supporting information

SUPPORTING INFORMATION

## Data Availability

The data that support the findings of this study are available for download from https://osf.io/n68fm.
