# Supplementary material for: Development of directed global inhibition, competitive inhibition and behavioural inhibition during the transition between infancy and toddlerhood
Source: Dev Sci. 2021 Dec 7;25(5):e13193. doi: 10.1111/desc.13193 (PMC11475536; doi:10.1111/desc.13193)
Supplement: Supplementary file 1 — SUPPORTING INFORMATION [file DESC-25-e13193-s001.docx]

Supplementary Materials 1 – Further Methods Detail

## ECITT coding scheme

The iPad recorded the accuracy and reaction time (RT; in milliseconds) of each response. These were later reviewed against video recordings for accuracy and validity. Trials were marked as invalid if one or more of the following behaviours were observed:

- The infant tapped both targets (the ‘buttons’ on the prepotent and inhibitory locations) with two hands simultaneously (i.e within the same frame)
- Reaction time <300ms (this occurs when the infant still had their hand in position from previous tap, or was already reaching before the new set of stimuli had been shown, so cannot be considered a response to the stimuli)
- Infant was clearly not attending and tapped by accident: Examples of accidental tapping include but are not limited to: looking at the researcher or parent when making a response; touched the screen when fussing, with head turned away from the iPad; fidgeting with the tape around the tablet; catching the screen with hand when moving the iPad or when the researcher moves the iPad. At 10 months this sometimes happens when the infant is trying to push/pull tablet away and then accidentally draws their hand across screen – thus there is a response, but it is not deliberately directed to a stimulus.
- Parent interference: Points/nudges or otherwise indicates which one to touch
- Researcher interference: Points/nudges or otherwise indicates which one to touch
- Reaching behaviour obscured: e.g., infant stood on parent’s lap, so screen and reach were out of range of all videos (multiple cameras were positioned around the room to limit this as much as possible).
- Infant used their parent’s hand or another part of their own body than their hands (e.g., nose, forehead, foot, mouth, elbow etc.) to make the response.
- Infant deliberately touches the middle of the screen (i.e. the gap exactly between buttons)

In cases where the first tap was not registered by the iPad, and the infant then changed their response to a different side, the accuracy of the first tap was coded and treated as valid (unless one of the above invalid conditions was also met). For accuracy analyses, all trials retained after the above exclusions were included.

For the 16-month visit, software-recorded RTs were verified against the video by trained coders. If the software did not register the first touch, this was corrected using RTs identified from the video to within 33ms accuracy. Trials with a RT longer than 5000ms were excluded from RT analysis only (these trials occurred when a child got distracted).

## A-not-B coding scheme

The infant’s behaviour during the A-not-B task was video-recorded and coded offline for accuracy and validity. Accuracy was assessed on the basis of the first touch of a well cover; e.g., on a B trial if an infant first touched cover A, but then pulled the cover off B, they would be scored as incorrect. Trials were marked as invalid if one or more of the following behaviours were observed:

- The infant did not attempt to retrieve the toy
- The infant did not attend to the hiding event
- The infant touched both covers simultaneously (i.e. within the same frame) and did not clearly orient to one side. Infants who touched both covers simultaneously but oriented with body and gaze to one, would be scored as valid and their accuracy rated according to the side that they had oriented to
- The infant tried to grab the toy before the hiding event
- The infant only touched the centre-point between the two wells (if they then made a reaching response to a well, the trial would be treated as valid).
- The infant’s touch was clearly accidental. If the infant was looking at the researcher when reaching but then orients to the box this would be coded as valid – an accidental reach is recorded only when the infant turned away (e.g. to orient to their parent) and caught the box by accident).
- The infant bashed repeatedly at the box without orienting to a well such that any touching of the covers appears accidental
- Parent interference: Points/nudges or otherwise indicates which one to touch
- Researcher interference: Points/nudges or otherwise indicates which one to touch
- Incorrect side administered
- Imperfect hiding event: covers not placed flat over the wells

## Missing data

Supplementary Table 1.1. Summary of missing and excluded data

| Task | Reason for exclusion | 10 months Session 1 | 10 months Session 2 | 16 months |
| --- | --- | --- | --- | --- |
| Touchscreen Approach | Not administered (not yet included in protocol) | 3 | - | 0 |
|  | No video for coding and validation checks | 2 | - | 0 |
|  | Refused to touch, even after demo and encouragement | 0 | - | 3 |
| Toy Prohibition | Not administered (not yet included in protocol) | 28 |  | 3 |
|  | Distressed before starting administration | 0 |  | 1 |
| Touchscreen Prohibition | No video for coding and validation checks | 5 | - | 0 |
|  | Technical issue (app crashed) | 1 | - | 0 |
|  | Administration error | 0 | - | 2 |
|  | Would not touch after prohibition released | 0 | - | 1 |
| A-not-B | Not administered (not included in protocol) | 23 | - | 0 |
|  | No video for coding and validation checks | 5 | - | 0 |
|  | Experimenter error | 0 | - | 2 |
|  | Fewer than 10 valid trials | 3 | - | 2 |
|  | Did not reach to each location at least once | 1 | - | - |
| ECITT | No video for coding and validation checks | 5 | 0 | 1 |
|  | Completed fewer than 10 valid trials and/or fewer than 2 inhibitory trials | 12 | 2 | 1 |
|  | Refused to attempt | 0 | 0 | 1 |
|  | Did not meet prepotent performance thresholds (minimum 60% correct) | 7 | 1 | 6 |

For the ECITT, the proportion of infants with missing data was 17.6% at 10 months (11.1% unable/unwilling to complete sufficient valid trials, 6.5% not meeting the pre-potent performance threshold), and 11.5% at 16 months (2.6% unable/unwilling to complete sufficient valid trials, 9.0% not meeting pre-potent performance threshold). For the A-not-B task, the proportion of infants with systematically missing data was 3.7% at 10 months (2.8% unable/unwilling to complete sufficient valid trials, .9% did not reach to each location at least once), and 2.6% at 16 months (unable/unwilling to complete sufficient valid trials).

# Supplementary Materials 2 – Sex differences

## Sex differences

As shown in Supplementary Table 2.1, no sex differences were observed for any of the tasks.

Supplementary Table 2.1. Sex differences in task performance at 10 and 16 months.

| Construct | Variable | Test statistic | *p* |
| --- | --- | --- | --- |
| Behavioural inhibition | Touchscreen Approach 10 months | *Z*=-.1.531 | .126 |
|  | Touchscreen Approach 16 months | *Z*=-.1.841 | .066 |
| Directed global inhibition | Touchscreen prohibition 10 months | *Z*=-1.063 | .290 |
|  | Touchscreen prohibition 16 months | *Z*=-0.571 | .572 |
|  | Toy prohibition 10 months | *Z*=-1.266 | .208 |
|  | Toy prohibition 16 months | *Z*=-0.300 | .770 |
| Competitive inhibition | ECITT AccD 10 months | *t*=0.854 | .394 |
|  | ECITT AccD 16 months | *t*=-0.391 | .696 |
|  | ECITT Switching 10 months | *t*=0.726 | .468 |
|  | ECITT Switching 16 months | *t*= -1.054 | .296 |
|  | A-not-B Accuracy 10 months | *t*= 0.099 | .921 |
|  | A-not-B Accuracy 16 months | *t*= 0.098 | .922 |
|  | A-not-B Switching 10 months | *Z=*-0.120 | .907 |
|  | A-not-B Switching 16 months | *Z=*-1.165 | .247 |

# Supplementary Materials 3 – Analyses using imputed data

Correlational analyses were re-run after imputing missing data. Following recommendations of Bodner (2008) and White at al. (2011), we matched the number of imputations (40) to the highest proportion of data that was missing on any variable (16-month ECITT AccD). We imputed data only where we were confident that data were missing at random – i.e. due to technical or protocol reasons (see Supplementary Table 1.1). Data that were excluded for child reasons (i.e. refused to attempt, or did not achieve validity criteria) or due to lack of follow-up were not imputed as we could not be confident that data were missing at random. Conclusions from correlations using imputed data were consistent with those reported in the main manuscript.

Supplementary Table 3.1. Bivariate associations between tasks at 10 months, using imputed data. Cells show the correlation coefficient, with *n* in italics. Variable names indicate the target construct in parentheses.

|  | Touchscreen prohibition (DGI) | Toy prohibition  (DGI) | A-not-B switching  (CI) | ECITT AccD  (CI) | ECITT Switching  (CI) |
| --- | --- | --- | --- | --- | --- |
| Touchscreen Approach (BI) | .306* | .194 | -.069 | .044 | .099 |
| Touchscreen prohibition (DGI) |  | .336* | -.165 | .219 | .131 |
| Toy prohibition  (DGI) |  |  | -.201 | .070 | .091 |
| A-not-B Accuracy (CI) |  |  |  | .053 | .050 |
| A-not-B switching (CI) |  |  |  | .177 (.155)^a^ | .082 (.050)^a^ |

BI: Behavioural Inhibition. DGI: Directed Global Inhibition. CI: Competitive Inhibition.

ECITT: Early Childhood Inhibitory Touchscreen Task; AccD: Accuracy Difference

*Significant after applying the Benjamini-Hochberg correction for 16 tests with an alpha of .05.  ^a^A-not-B switching correlations presented for Pearson’s with Spearman’s *rho* in brackets, due to high skew.

Exploratory tests use Spearman’s *rho* used for analyses involving skewed Touchscreen Approach and both Prohibition variables, and Pearson’s for all other correlations.
Pre-registered tests (underlined) are 1-tailed, all other tests 2-tailed. *n* for each test = 90-103

Supplementary Table 3.2. Bivariate associations between tasks at 16 months, using imputed data. Cells show the correlation coefficient. Variable names indicate the target construct in parentheses.

|  | Touchscreen prohibition (DGI) | Toy prohibition  (DGI) | A-not-B switching  (CI) | ECITT AccD  (CI) | ECITT Switching  (CI) |
| --- | --- | --- | --- | --- | --- |
| Touchscreen Approach (BI) | .129 | -.076 | .114 | .207 | -.019 |
| Touchscreen prohibition (DGI) |  | .598* | .075 | .144 | -.108 |
| Toy prohibition (DGI) |  |  | -.011 | .059 | .035 |
| A-not-B switching (CI) |  |  |  | .437* (.430)^a^ | .305* (.171)^a^ |

BI: Behavioural Inhibition. DGI: Directed Global Inhibition. CI: Competitive Inhibition.

ECITT: Early Childhood Inhibitory Touchscreen Task; AccD: Accuracy Difference

*Significant after applying the Benjamini-Hochberg correction for 14 tests with an alpha of .05.  ^a^A-not-B switching correlations presented for Pearson’s with Spearman’s *rho* in brackets, due to high skew.

Exploratory tests use Spearman’s *rho* for analyses involving skewed Touchscreen Approach and both Prohibition variables, and Pearson’s for all other correlations.
Pre-registered tests (underlined) are 1-tailed, all other tests 2-tailed. *n* for each test = 67-76

Supplementary Table 3.3. Bivariate correlations between behavioural inhibition and inhibitory control measures at 10- and 16-months, using data imputed cross-sectionally. Cells show the correlation coefficient. Variable names indicate the target construct in parentheses.

|  | 16m Touchscreen Approach (BI) | 16m Touchscreen prohibition (DGI) | 16m Toy prohibition (DGI) | 16m ECITT AccD (CI) | 16m ECITT Switching (CI) | 16m A-not-B switching (CI) |
| --- | --- | --- | --- | --- | --- | --- |
| 10m Touchscreen Approach (BI) | - | -.109 | -.145 | .235 | .288 | -.038 |
| 10m Touchscreen prohibition (DGI) | .042 | - | .104 | .083 | .179 | .002 |
| 10m Toy prohibition (DGI) | -.150 | -.003 | - | .255 | .133 | -.007 |
| 10m ECITT AccD (CI) | -.113 | .246 | .143 | - | - | - |
| 10m ECITT Switching (CI) | -.025 | .064 | .025 | - | - | -.175 (-.224) |
| 10m A-not-B switching (CI) | .229 | -.009 | -.120 | -.068(-.064) | .033 (.041) | - |

BI: Behavioural Inhibition. DGI: Directed Global Inhibition. CI: Competitive Inhibition.

M: month; ECITT: Early Childhood Inhibitory Touchscreen Task; AccD: Accuracy Difference

A-not-B and ECITT switching correlations presented for Pearson’s with Spearman’s rho in brackets, due to high skew. Spearman’s rho used for analyses involving skewed Touchscreen Approach and both Prohibition variables, and Pearson’s for ECITT AccD correlations.
Pre-registered tests (underlined) are 1-tailed, all other tests 2-tailed. *n* for each test = 56-67

Supplementary Table 3.4. Bivariate correlations between behavioural inhibition and inhibitory control measures at 10- and 16-months, using data imputed cross-sectionally at 10 months. Cells show the correlation coefficient. Variable names indicate the target construct in parentheses.

|  | 16m Touchscreen Approach (BI) | 16m Touchscreen prohibition (DGI) | 16m Toy prohibition (DGI) | 16m ECITT AccD (CI) | 16m ECITT Switching (CI) | 16m A-not-B switching (CI) |
| --- | --- | --- | --- | --- | --- | --- |
| 10m Touchscreen Approach (BI) | *-* | -.109  *67* | -.145  *67* | .205  *64* | .277  *59* | -.038  *66* |
| 10m Touchscreen prohibition (DGI) | .042  67 | - | .104  *67* | .054  *64* | .220  *58* | .002  *66* |
| 10m Toy prohibition (DGI) | -.150  *66* | -.003  *67* | *-* | -.088  *64* | .133  *59* | -.007  *66* |
| 10m ECITT AccD (CI) | -.113  *56* | .246  *57* | .143  *57* | - | *-* | - |
| 10m ECITT Switching (CI) | -.025  *56* | .064  *57* | .025  *57* | - | *-* | -.175(-.224)  *56* |
| 10m A-not-B switching (CI) | .229  *62* | -.009  *63* | -.120  *63* | -.039(-.049)  *62* | .036(.045)  *55* | - |

BI: Behavioural Inhibition. DGI: Directed Global Inhibition. CI: Competitive Inhibition.

M: month; ECITT: Early Childhood Inhibitory Touchscreen Task; AccD: Accuracy Difference

A-not-B and ECITT switching correlations presented for Pearson’s with Spearman’s rho in brackets, due to high skew. Spearman’s rho used for analyses involving skewed Touchscreen Approach and both Prohibition variables, and Pearson’s for ECITT AccD correlations.
Pre-registered tests (underlined) are 1-tailed, all other tests 2-tailed. *n* for each test = 56-67

# Supplementary Materials 4 –Additional analyses

## 4.1 Side bias

At 10 months (session 1), independent *t*-tests indicated that infants had significantly lower ECITT AccD scores when the prepotent side was on the right (*M*=.538, *SD*=.298), compared to the left (*M*=.752, *SD*=.352) (*t*(82) = 3.009, *p*=.003, *d* =.657). Similarly, at 16 months, infants had significantly lower ECITT AccD scores when the prepotent side was on the right (*M*=-.532, *SD*=.364), compared to the left (*M*=.736, *SD*=.358) (*t*(64) = 2.277, *p*=.026, *d* =.565). However, ECITT switching scores were not significantly affected by prepotent side at 10 (*t*(85.17) = 0.479, *p*=.633, *d* =.099) or 16 months (*t*(42.283) = 0.589, *p*=.559, *d* =.155).

## 4.2 Spill-over effects (pre-registered exploratory analyses)

There was no significant difference in A-not-B switching performance depending on whether the ECITT prepotent side (used as the side for ‘B’ trials) was on the left or right at either 10 (*Z*=-1.207, *p*=.227) or 16 months (*Z*=-1.864, *p*=.062). Nor was accuracy on the first A-not-B trial significantly associated with ECITT side at 10 (Χ^2^(1)=0.002, *p*=1.000) or 16 months (Χ^2^(1)=1.158, *p*=.337). This indicates that there were not spill-over effects from ECITT to A-not-B.

## 4.3 Test-retest of performance on inhibitory and prepotent ECITT trials

Pearson’s correlations indicated a significant moderate positive association between ECITT IAcc scores at the test and re-test visits (*r*=.496, *p*<.001), and a non-significant weak positive association between ECITT PAcc scores at the test and re-test visits (*r*=.247, *p*=.090).

Paired *t*-tests indicated no significant differences in means between test and re-test ECITT IAcc scores (*t*(47) = -1.686, *p*=.098, *d* =.243) or PAcc scores (*t*(47) = 0.137, *p*=.891, *d* =.020).

Paired *t*-tests found no significant differences in means between test and re-test ECITT AccD (*t*(47)=-1.347, *p*=.184, *d*=.194) or Switching scores (*t*(50)=-1.057, *p*=.296, *d*=.148), indicating that infants did not show a significant training effect between Sessions 1 and 2. Nevertheless, only scores from Session 1 are used in the analyses below and in the main manuscript.

## 4.4 Exclusions of extreme scores (pre-registered supplementary analyses)

Visual inspection of histograms and Q-Q plots indicated that ECITT and A-not-B data approximated a normal distribution, with the exception of A-not-B switching scores and 16 month ECITT switching scores – for which we also report Spearman’s *rho* correlation coefficients, in addition to Pearson’s. In accordance with the pre-registrations, we also considered the effect of excluding extreme values more than 2 standard deviations above or below the mean (note that 16 month ECITT switching scores are normally distributed after excluding extreme scores).

Supplementary Table 4.4.1. Exclusions to ECITT and A-not-B data made on the basis of extreme values

| Variable | Number more than 2SD below mean | Number more than 2SD above mean |
| --- | --- | --- |
| ECITT AccD 10m | 0 | 1 |
| ECITT AccD 16m | 0 | 0 |
| ECITT Switching 10m | 2 | 2 |
| ECITT Switching 16m | 1 | 2 |
| A-not-B Accuracy 10m | 0 | 0 |
| A-not-B Switching 10m | 0 | 5 |
| A-not-B Switching 16m | 0 | 2 |

ECITT: Early Childhood Inhibitory Touchscreen Task; AccD: Accuracy Difference; SD: Standard deviation.

Supplementary Table 4.4.2 Bivariate associations between tasks at 10 months, excluding ECITT and A-not-B observations more than 2 standard deviations above or below the mean. Cell values show the correlation coefficient, and *n* in italics. Pre-registered tests are underlined.

|  | A-not-B switching | ECITT AccD | ECITT Switching |
| --- | --- | --- | --- |
| Touchscreen Approach | -.056  *71* | ­.055  *83* | .129  *87* |
| Touchscreen prohibition | -.232  *71* | .174  *83* | .119  *86* |
| Toy prohibition | -.223  *55* | .098  *69* | .041  *71* |
| A-not-B Accuracy | - | .046  *62* | -.027  *64* |
| A-not-B switching | - | .195 (.186)^a^  *59* | .053 (.059) ^a^  *60* |

No tests were significant after applying the Benjamini-Hochberg correction for 16 tests (see Table 5) with an alpha of .05.
^a^A-not-B switching correlations presented for Pearson’s with Spearman’s *rho* in brackets, due to high skew

ECITT: Early Childhood Inhibitory Touchscreen Task; AccD: Accuracy Difference

Spearman’s *rho* used for analyses involving skewed Approach and Prohibition variables, and Pearson’s for all other correlations. Pre-registered tests are 1-tailed, all other tests 2-tailed.

Supplementary Table 4.4.3*.* Bivariate associations between tasks at 16 months, excluding ECITT and A-not-B observations more than 2 standard deviations above or below the mean. Cell values show the correlation coefficient, and *n* in italics. Pre-registered tests are underlined.

|  | A-not-B switching | ECITT AccD | ECITT Switching |
| --- | --- | --- | --- |
| Touchscreen Approach | .170  *69* | .207 | -.054  *69* |
| Touchscreen prohibition | .076  *70* | .157 | -.059  *68* |
| Toy prohibition | .031  *71* | .092 | .056  *66* |
| A-not-B switching |  | .412* (.411)^a^  *63* | .050 (.009)^a^  *65* |

*Significant after applying the Benjamini-Hochberg correction for 14 tests (see Table 6) with an alpha of .05
^a^ A-not-B switching correlations presented for Pearson’s with Spearman’s *rho* in brackets, due to high skew

ECITT: Early Childhood Inhibitory Touchscreen Task

Spearman’s *rho* used for analyses involving Approach and Prohibition variables, and Pearson’s for all other correlations. Pre-registered tests are 1-tailed, all other tests 2-tailed.

Supplementary Table 4.4.4. Changes in 10- and 16-month-olds’ ECITT switching performance, excluding observations more than 2 standard deviations above or below the mean.

| Task | Developmental progression | | | | Longitudinal stability | | | |
| --- | --- | --- | --- | --- | --- | --- | --- | --- |
|  | Test statistic | *p* | Effect size (d) | Correlation coefficient | | Confidence interval | n |  |
| ECITT Switching | *t=-8.141* | *<.001* | 1.200 | .453* | | .207, .638 | *46* |  |

ECITT: Early Childhood Inhibitory Touchscreen Task;
*Significant after applying the Benjamini-Hochberg correction for 6 tests (see Table 7) with an alpha of .05
1-tailed, the pre-registered Pearson’s correlation is underlined.

Supplementary Table 4.4.5. Bivariate correlations between behavioural inhibition and inhibitory control measures at 10- and 16-months, excluding ECITT and A-not-B observations more than 2 standard deviations above or below the mean. Cell values show the correlation coefficient, and *n* in italics.

|  | 16m A-not-B switching | 16m ECITT Switching |
| --- | --- | --- |
| 10m Touchscreen Approach | -.096  *64* | *.*265  *60* |
| 10m Touchscreen prohibition | .091  *63* | .202  *59* |
| 10m Toy prohibition | .023  *49* | .132  *47* |
| 10m A-not-B switching | - | .113 (.088)  *38* |

M: month; ECITT: Early Childhood Inhibitory Touchscreen Task

Spearman’s *rho* used for analyses involving skewed Approach and Prohibition variables, and Pearson’s for all other correlations. Pre-registered tests are 1-tailed, all other tests 2-tailed. The pre-registered Pearson’s correlation is underlined.

## 4.5 Excluding repeat inhibitory trials (pre-registered exploratory analyses)

As shown in Supplementary Tables 4.5.1-4.5.4, conclusions regarding longitudinal stability, developmental progression, and associations between tasks were unaffected by restricting ECITT AccD scores to the first inhibitory trial in a row.

Supplementary Table 4.5.1. Changes in ECITT AccD scores between 10 and 16 months when repeat inhibitory trials were excluded

| Task | Developmental progression | | | | Longitudinal stability | | | |
| --- | --- | --- | --- | --- | --- | --- | --- | --- |
|  | Test statistic | *p* | Effect size (d) | Correlation coefficient ^a^ | | Confidence interval | n |  |
| ECITT AccD | *t* = 1.659 | .105 | .256 | .008 | | -.304, .331 | 42 |  |

Supplementary Table 4.5.2. Bivariate associations between tasks at 10 months, restricting ECITT AccD scores to the first inhibitory trial in a row. Cell values show the correlation coefficient, and *n* in italics.

|  | ECITT AccD excluding repeat inhibitory trials |
| --- | --- |
| Touchscreen Approach | .040  *74* |
| Touchscreen prohibition | .146  *74* |
| Toy prohibition | -.035  *62* |
| A-not-B Accuracy | .139  *53* |
| A-not-B switching | -.171(-.185)^a^  *53* |

^a^A-not-B switching correlations presented for Pearson’s with Spearman’s *rho* in brackets, due to high skew

ECITT: Early Childhood Inhibitory Touchscreen Task; AccD: Accuracy Difference

Exploratory tests use Spearman’s *rho* for analyses involving skewed Approach and Prohibition variables, and Pearson’s for all other correlations. Pre-registered tests (underlined) are 1-tailed, all other tests 2-tailed.

Supplementary Table 4.5.3. Bivariate associations between tasks at 16 months, restricting ECITT accuracy score to the first inhibitory trial in a row. Cell values show the correlation coefficient, and *n* in italics.

|  | ECITT AccD excluding repeat inhibitory trials |
| --- | --- |
| Touchscreen Approach | .186  *66* |
| Touchscreen prohibition | .139  *66* |
| Toy prohibition | .051  *64* |
| A-not-B switching | .453* (.441)^a^  *64* |

*Significant after applying the Benjamini-Hochberg correction for 14 tests with an alpha of .05.  ^a^ A-not-B switching correlations presented for Pearson’s with Spearman’s *rho* in brackets, due to high skew

ECITT: Early Childhood Inhibitory Touchscreen Task; AccD: Accuracy Difference

Exploratory tests use Spearman’s *rho* for analyses involving skewed Approach and Prohibition variables, and Pearson’s for all other correlations. Pre-registered tests (underlined) are 1-tailed, all other tests 2-tailed.

Supplementary Table 4.5.4. Bivariate associations between performance on ECITT at 16 months, restricting ECITT accuracy score to the first inhibitory trial in a row, and behavioural inhibition and inhibitory control measures at 10 months. Cell values show the correlation coefficient, and *n* in italics.

|  | 16m ECITT AccD excluding repeat inhibitory trials |
| --- | --- |
| 10m Touchscreen Approach | .233  *59* |
| 10m Touchscreen prohibition | .080  *58* |
| 10m Toy prohibition | .242  *46* |
| 10m A-not-B switching | -.004 (.012)  *41* |
| 10m ECITT AccD | -.008  *42* |

^a^ A-not-B switching correlations presented for Pearson’s with Spearman’s *rho* in brackets, due to high skew

ECITT: Early Childhood Inhibitory Touchscreen Task; AccD: Accuracy Difference

Exploratory tests use Spearman’s *rho* for analyses involving skewed Approach and Prohibition variables, and Pearson’s for all other correlations. Pre-registered tests (underlined) are 1-tailed, all other tests 2-tailed.
